# Safety, Feasibility, and User Experience of Automated Insulin Delivery Systems During Hajj (Muslim Pilgrimage)

**DOI:** 10.3390/jcm15020860

**Published:** 2026-01-21

**Authors:** Mohammed E. Al-Sofiani

**Affiliations:** 1 Division of Endocrinology, Diabetes and Metabolism, College of Medicine, King Saud University, Riyadh 12372, Saudi Arabia; malsofiani@ksu.edu.sa; 2 Division of Endocrinology, Diabetes & Metabolism, Johns Hopkins University, Baltimore, MD 21287, USA; 3 Strategic Center for Diabetes Research, College of Medicine, King Saud University, Riyadh 12372, Saudi Arabia

**Keywords:** automated insulin delivery, Hajj, pilgrimage, type 1 diabetes

## Abstract

**Background/Objectives:** Performing Hajj, the annual Islamic pilgrimage to Mecca and one of the world’s largest mass gatherings, involves considerable physical exertion in high temperatures and presents unique challenges for people with type 1 diabetes (PWT1D). We examined the feasibility, safety, and user experience of automated insulin delivery (AID) systems during Hajj. **Methods:** This mixed-methods study evaluated six PWT1D who used an AID pump (2 MiniMed 780G, 2 Medtrum, 1 OmniPod 5, and 1 Open-source AID) while performing Hajj in 2024–2025. Pump and CGM-derived metrics were compared across pre-Hajj, during Hajj, and post-Hajj periods. A structured survey captured participants’ experiences, challenges, and recommendations for AID use during Hajj. **Results:** The average percent time in range (TIR) remained stable from pre- to during Hajj (54.98 to 54.18, *p* > 0.05) and significantly increased post-Hajj (62.62, *p* < 0.05). The percent time above range (TAR > 180) and Glycemia Risk Index significantly decreased from pre- to post-Hajj (28.34 to 26.28 and 50.3 to 19.3, respectively, both *p* < 0.05). The percent time below range (TBR) remained low (<1%) across the three periods with no incidence of acute diabetes-related complications. Participants emphasized increased confidence and peace of mind with AID use and reported challenges related to heat exposure, prolonged walking, and lack of awareness regarding diabetes technology among HCPs. **Conclusions:** The use of AID during Hajj appeared to be safe and effective for PWT1D in our study, maintaining stable glycemic control under physically demanding conditions. As the first study to evaluate AID use during Hajj, our findings call for larger studies to explore the integration of diabetes technology into Hajj care protocols and highlight the need for structured pre-Hajj education for PWT1D and HCPs.

## 1. Background

A substantial proportion of people with diabetes (PWD) live in Muslin-majority countries, particularly within the Middle East and North Africa (MENA) region. According to the latest International Diabetes Federation (IDF) Atlas, the MENA region has one of the world’s highest diabetes prevalence rates, with approximately one in six adults affected [1]. Likewise, several Muslim-majority countries outside the MENA region, including Indonesia, Bangladesh, Turkey, and Pakistan, also report high rates of diabetes [1]. Considering these figures, religious practices such as Ramadan fasting and Hajj pilgrimage hold considerable public health importance in the context of diabetes management.

**Figure 1:**
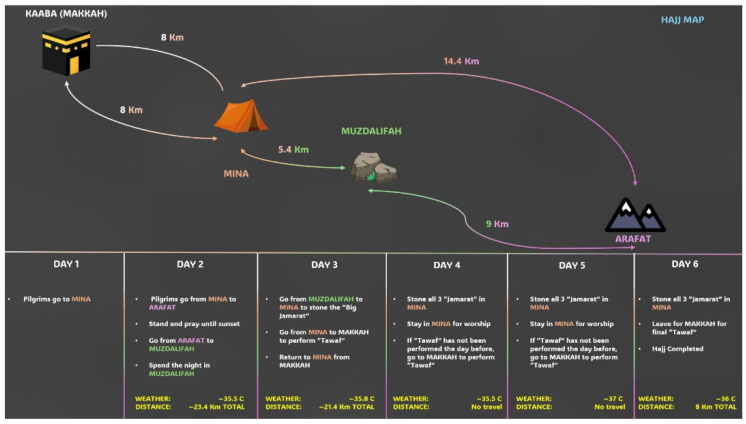
Chronological sequence of Hajj rituals, pilgrim movements, and physical activities.

Hajj, the Islamic pilgrimage to Mecca, is one of the five pillars of Islam and constitutes one of the world’s largest annual mass gatherings worldwide. Each year, approximately 1.5 to 2.5 million Muslims from more than 180 countries travel to Mecca, spending 5 to 15 days in multiple ritual sites (Figure 1) [2]. Hajj involves considerable physical exertion, including extensive walking for long distances in high temperatures, often compounded by overcrowding, irregular meal patterns, and intermittent access to water. As a result, pilgrims are at increased risk of dehydration, heat stroke, and infectious diseases.

For PWD, performing Hajj carries additional potential risks, including severe hypoglycemia, hyperglycemia, diabetic ketoacidosis (DKA), hyperosmolar hyperglycemic state (HHS), and foot injury [3,4,5]. Those using insulin, including people with type 1 diabetes (PWT1D), are considered moderate to very high risk during Hajj, depending on their glycemic control and other factors [6]. Yet, they represent a substantial proportion of pilgrims each year. There are currently no published studies evaluating the effectiveness or safety of specific diabetes management approaches during Hajj. This literature gap limits healthcare professionals’ (HCPs’) ability to provide evidence-based counseling and risk mitigation strategies for pilgrims with diabetes. To our knowledge, no previous studies have assessed the safety and performance of automated insulin delivery (AID) systems in this unique setting.

In this study, we evaluated the feasibility and safety profiles of four different AID systems in PWT1D who performed Hajj. In addition, we conducted an in-depth qualitative assessment of participants’ experience to gain insights into their diabetes management during Hajj, identify key challenges encountered, and explore potential strategies to enhance safety and support for insulin pump users during Hajj.

## 2. Research Design and Methods

### 2.1. Participants

We included six adults with T1D who used an AID system and were under the care of Dr. Mohammed Al-Sofiani at MyClinic Center in Riyadh, Saudi Arabia. All participants performed Hajj during 2024 or 2025 and provided consent to share their Hajj experience and feedback. We retrospectively reviewed participants’ pump and CGM reports following the completion of Hajj.

### 2.2. Outcomes and Covariates

The primary outcome was the change in time in range (TIR), defined as 70–180 mg/dL (3.9–10 mmol/L) across the following three predefined periods:

(1) Pre-Hajj week: 26 May–2 June 2025;
(2) During Hajj week: 3–10 June 2025;
(3) Post-Hajj week: 11–18 June 2025.

For the one participant who performed Hajj in 2024, the corresponding comparison periods were as follows:

(1) Pre-Hajj week: 4–11 June 2024;
(2) During Hajj week: 12–19 June 2024;
(3) Post-Hajj week: 20–27 June 2024.

Secondary outcomes included changes in the following:

- Time below range (TBR): both Level 1 (TBR < 70 mg/dL; <3.9 mmol/L) and Level 2 (<54 mg/dL; <3 mmol/L);
- Time above range (TAR): both Level 1 (TAR > 180; >10 mmol/L) and Level 2 (>250; >13.9 mmol/L);
- Glycemia Risk Index (GRI);
- Coefficient of variation (CV);
- Insulin total daily dose (TDD) and the relative percentages of basal and bolus insulin within TDD.

### 2.3. Qualitative Evaluation

For the qualitative part of this mixed-methods study, a structured Arabic-language online survey was sent out to the six participants. The aim of the survey was to gain a deeper understanding of participants’ Hajj experiences, diabetes self-management during Hajj, and their perceptions of using AID in this unique context. The survey also aimed to identify key challenges, potential solutions, and recommendations for improving diabetes care during Hajj.

The survey included a combination of multiple-choice questions (MCQs) and open-ended questions to allow for both quantitative and qualitative insights. The survey had three main sections:

*Section 1*: Participant characteristics: Including age, gender, duration of diabetes, type of AID system, and previous Hajj experience.

*Section 2*: Diabetes During Hajj: Addressing preparation for Hajj—perceived glucose control, diabetes-related complications during Hajj, the role of AID systems during Hajj, and access to healthcare services and pump and CGM supplies.

*Section 3*: Challenges and Recommendations: Exploring challenges encountered during Hajj, AID-related challenges, participants’ suggestions for improving Hajj experiences in the future.

The participants’ responses were collected electronically after Hajj and translated from Arabic to English for analysis. The translated responses were reviewed for accuracy by a bilingual person living with T1D who is familiar with the cultural and clinical context. We analyzed the qualitative data and identified recurrent themes using an inductive thematic analysis approach. Responses were first coded to capture salient concepts related to participants’ experiences, challenges, and perception of AID use during Hajj. Recurrent themes were identified and used to organize the results. Representative quotations were selected to illustrate each major theme. The study was approved by the Institutional Review Board at King Saud University (Approval No. E-25-9975), and informed consent was obtained from all participants prior to study participation.

### 2.4. Statistical Analysis

All significant testing was 2-tailed with α of 0.05, and data were analyzed using Stata Statistical Software (release 15). Categorical variables were presented as frequencies and percentages, whereas continuous variables were presented as means ± standard deviations (SDs). When comparing the CGM and pump metrics across the three study periods (pre-Hajj, during Hajj, and post-Hajj), a paired t-test was used to determine whether values during Hajj or after Hajj differed significantly from the pre-Hajj measurements.

## 3. Results

### 3.1. Participant Characteristics

Six individuals with T1D performed Hajj (five females and one male). Five participants had their pump and CGM reports shared with us, while one was unable to share the report due to technical difficulties. The average age of participants and duration of diabetes were 33 and 18 years, respectively (Table 1). The AID systems used by the six participants were two Medtronic 780G pumps, two Medtrum pumps, one OmniPod 5, and one Open-source AID (i.e., loop) system. All participants were performing Hajj for the first time.

### 3.2. Changes in Glycemic Control and Insulin Doses from Pre- to During Hajj

**Figure 2:**
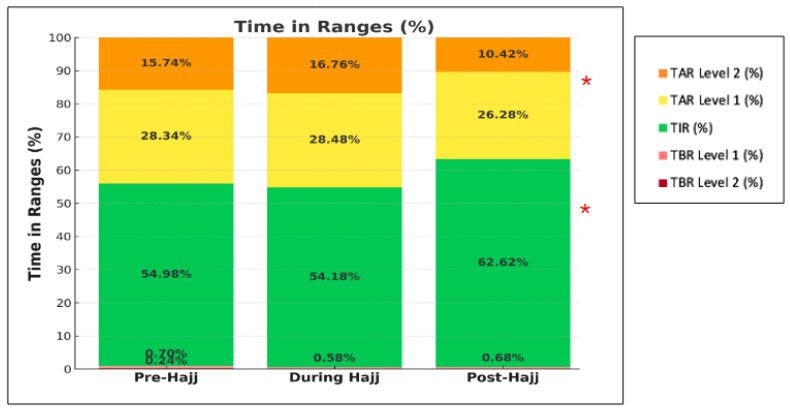
Changes in TIRs across pre-, during, and post-Hajj periods (*n* = 5). * *p* < 0.05 comparing the post-Hajj to the pre-Hajj.

There were no significant changes in any of the CGM-derived metrics from pre- to during Hajj (average TIR: 54.98 to 54.18%; TBR < 70: 0.70 to 0.58%; TBR < 54: 0.24 to 0%; TAR > 180: 28.34 to 28.48%; and TAR > 250: 15.74 to 16.76%; all *p* > 0.05) (Figure 2). Likewise, the GRI did not change significantly from pre- to during Hajj (50.3 to 51, *p* > 0.05) (Table 2). The TDD of insulin and the proportions of basal and bolus insulin also remained relatively stable from pre- to during Hajj (Table 2).

**Figure 3:**
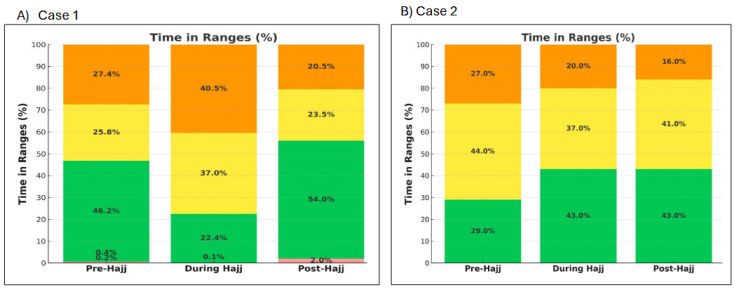

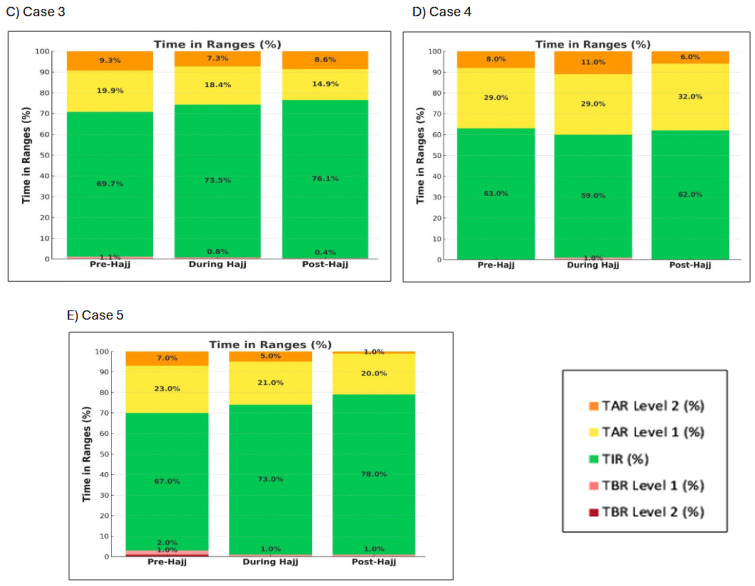
Changes in TIRs across pre-, during, and post-Hajj periods for each of the five cases.

**Figure 4:**
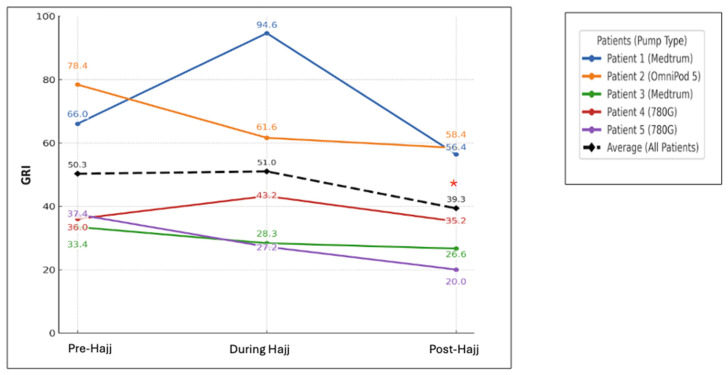
Changes in GRIs across pre-, during, and post-Hajj periods for each of the five cases. * *p* < 0.05 comparing the post-Hajj to the pre-Hajj values.

At the individual level, TIR increased during Hajj in all participants except cases 1 and 4. In case 1 (Medtrum user), TIR decreased from 46.2% pre-Hajj to 37% during Hajj, accompanied by an increase in TAR from 53.2% to 77.5% (Figure 3A) and an increase in GRI from 66 to 94.6 (Figure 4). It is worth noting that this patient reported decreased insulin efficacy on the second day of Hajj (Day of Arafat), when automated correction doses appeared less effective. In case 4 (Medtronic 780G user), TIR decreased from 63% pre-Hajj to 59% during Hajj, and GRI increased from 36 to 43.2 during Hajj (Figure 4). In all five participants, TBR remained very low throughout the three study periods (pre-, during Hajj, and post-Hajj) (Figure 3A–E).

### 3.3. Changes in Glycemic Control and Insulin Doses from Pre-Hajj to Post-Hajj

There was a statistically significant increase in average TIR from pre-Hajj to post-Hajj (54.98 to 62.62%, respectively, *p* < 0.05) and a significant decrease in TAR > 180 (28.34 to 26.28%, respectively, *p* < 0.05). There were no significant changes observed in the other CGM-derived metrics across the two periods (Figure 2). The GRI decreased significantly from pre- to post-Hajj (50.3 to 39.3, respectively, *p* < 0.05) (Table 2). The total daily dose (TDD) of insulin decreased from an average of 47.57 units pre-Hajj to 42.74 units post-Hajj. However, the relative percentages of basal and bolus insulin remained relatively stable (Table 2).

At the individual level, the TIR either increased or remained relatively the same from pre- to post-Hajj in all five cases, with some demonstrating marked improvement in TIR post-Hajj (Figure 3A–E).

### 3.4. Participant Experiences and Feedback About T1D and Hajj

All participants reported stable glucose levels during Hajj, with only one participant reporting symptomatic hypoglycemia that required oral carbohydrate intake (juice). Two participants adjusted their pump settings in preparation for Hajj, whereas others maintained their standard settings.

All participants emphasized the value of flexibility, peace of mind, and confidence afforded by AID systems during Hajj. They appreciated the automation of basal insulin delivery and the automated insulin delivery suspension before hypoglycemia occurs. The main difficulties reported by AID users during Hajj included prolonged walking, high temperatures, limited access to refrigeration for insulin storage, and irregular meal patterns requiring frequent snacking instead of structured meals. All participants had to prepare thoroughly for Hajj by meeting with their endocrinologist and carrying backup insulin and spare supplies for both their pump and CGM devices (Table 3).

Participants identified major gaps in diabetes technology awareness among healthcare personnel providing care in Hajj. One participant reported that an HCP was unable to differentiate between a CGM and an insulin pump, while another shared that an HCP incorrectly advised against the use of basal insulin injection following a pump infusion site failure in Arafat. The patient had no infusion tube replacement available at the time and kept asking the HCP to give her a basal insulin pen that she could use until she got back to Mina, where she kept the spare pump supplies. One participant stated the following: “*We need HCPs specialized in diabetes to be available and accessible during Hajj. It would be great to provide pilgrims with diabetes with digital wristbands that include medical information and connect to emergency teams if needed*”.

Overall, participants emphasized that adequate preparation and planning for Hajj are essential for a safe and successful Hajj. All participants recommended that PWT1D should be offered an insulin pump to use during Hajj and continue their use thereafter to maintain optimal glycemic control.

## 4. Discussion

In this first real-world pilot study evaluating the use of AID in six PWT1D during Hajj, we comprehensively assessed the feasibility, safety, and user experiences associated with AID use in this unique setting. Our findings demonstrate that AID systems maintained stable, and in some cases improved, glucose levels during and after Hajj compared with pre-Hajj levels. The significant increase in TIR and decrease in both GRI and TAR > 180 post-Hajj compared to pre-Hajj highlight the adaptability and effectiveness of AID systems in managing glucose fluctuations under conditions of intense physical activity, irregular routines, and extreme environmental temperatures. The absence of acute diabetes-related complications, such as severe hypoglycemia, DKA, and ER visits, further supports the safety of AID systems during Hajj. Additionally, the modest reduction in insulin requirement (i.e., TDD) during Hajj likely reflects an improved insulin sensitivity because of prolonged walking and increased physical exertion. This change is less likely to have been driven by changes in dietary patterns, as the relative proportions of basal to bolus insulin remained consistent across the pre-Hajj, during Hajj, and post-Hajj periods.

Our approach to preparing PWT1D using AID for Hajj includes proactive planning and patient education to ensure safety and uninterrupted insulin delivery during Hajj. Prior to travel, patients are advised to carry a sufficient supply of insulin, CGM sensors, and pump consumables. To minimize the risk of loss or damage, patients are advised to distribute their diabetes supplies across different bags. We also recommend that patients carry a portable cooling device for insulin storage. In addition, all patients traveling to Hajj should be provided with a glucagon emergency kit, with their companion trained on its administration. Furthermore, we recommend that patients carry a ketone meter with them for on-site monitoring during Hajj. In most cases, we did not need to change the pump setting in preparation for Hajj. However, we trained AID users on how to activate the temporary target (or exercise mode) during periods of increased physical activity (e.g., before, during, and after extended walking or when glucose levels approach 80 mg/dL and are trending down. An important lesson learned from this study is the necessity of keeping extra infusion sets and CGM sensors readily available at all times during Hajj. One participant experienced an infusion set failure in Arafat but was unable to return to Mina, where the backup supplies were stored, highlighting the importance of carrying spare equipment while performing the rituals. Table 4 summarizes key practical recommendations for clinicians and PWT1D using AID systems during Hajj.

The lack of awareness regarding diabetes technology among non-specialized HCPs was identified by PWT1D in our study as a major concern. This issue has been reported globally, but its consequences can be particularly serious in settings where healthcare resources are stretched [7,8]. One participant described a situation in which an HCP refused to administer basal insulin following an infusion site failure because a replacement infusion set was unavailable. Had the patient not insisted on receiving the injection, she could have developed DKA, a potentially life-threatening condition, particularly in resource-stretched healthcare environments such as those encountered during Hajj. These findings highlight the urgent need for national educational initiatives aimed at enhancing the knowledge and preparedness of non-specialized HCPs regarding diabetes technologies, both during Hajj and in routine clinical practice.

Our findings have important clinical and public health implications. To date, there have been no published studies evaluating the effectiveness and safety of specific diabetes therapies during Hajj. This resulted in a lack of evidence-based guidance for HCPs and PWD going for Hajj. The sustained and, in some cases, improved glycemic control observed among AID users in our study during and after Hajj underscores the potential of these systems to support safe diabetes management under demanding conditions. Several environmental and behavioral factors unique to Hajj may have influenced the glycemic outcomes and the performance of AID systems. These include prolonged periods of walking and physical exertion, exposure to high temperatures, increased risk of dehydration, and irregular meal timing and composition. Such factors are known to affect glycose dynamics and insulin sensitivity. Nonetheless, our findings are consistent with other studies evaluating the performance of AID systems during strenuous physical activities outside the context of Hajj [9,10]. For example, Jayawardene et al. reported that AID systems were able to maintain acceptable glucose levels and reduce the risk of hypoglycemia from high-intensity exercise [10]. Our results may encourage the broader adoption of AID technology across Muslim-majority countries, where utilization remains among the lowest worldwide despite a high burden of diabetes [11]. Expanding access to AID systems and providing structured pre-Hajj education could help optimize outcomes for PWD participating in Hajj and other physically demanding religious observances.

Our study has several strengths, most notably being the first published study evaluating the effectiveness and safety of multiple AID systems during Hajj. In addition to objective glycemic data, we also evaluated the patients’ perspectives and insights, providing a comprehensive understanding of the user experience in this unique and demanding setting. However, several limitations of our study should be acknowledged. The small sample size limits statistical power; however, our novel case series establishes a foundation and rationale for larger studies aimed at exploring diabetes technology use in high-risk PWD during Hajj. Moreover, our study participants were managed by a multidisciplinary team of an endocrinologist, diabetes educator, and dietitian with experience in diabetes technology, which may not reflect the standard of care in many regions and may limit the generalizability of our findings. Finally, we did not collect data on the socio-economic background, dietary intake, physical activity, or hydration levels of the study participants. Considering that our study participants likely had above-average access to healthcare resources, our findings may not be fully generalizable to the broader population of PWT1D or to less resourced healthcare settings.

## 5. Conclusions

In this first real-world evaluation of AID systems during Hajj, we demonstrated that the AID use in our patients with T1D was both safe and feasible for PWT1D under the physically demanding and resource-constrained conditions of Hajj. Most AID users had stable or improved glucose levels during and post-Hajj without an increase in hypoglycemia or acute diabetes-related complications. Participants reported high satisfaction, confidence, and peace of mind with AID use despite several gaps in HCP awareness about diabetes technology and logistical challenges related to health exposure and supply accessibility. The results of this study may help establish a new use of advanced diabetes technology and expand access to AID systems, particularly in Muslim-majority countries. Future larger-scale, multicenter studies are needed to compare the effectiveness and safety of different treatment modalities of T1D during Hajj.

## Figures and Tables

**Table 1:** Characteristics of participants (*n* = 6).

	All (*n* = 6)	Case 1	Case 2	Case 3	Case 4	Case 5	Case 6
Age, mean ± SD, years	33 ± 3.7	33	26	34	36	32	36
Gender, *n* (%)	5 Female (83%), 1 Male (17%)	F	M	F	F	F	F
Diabetes Duration, mean ± SD, years	18 ± 8.2	4	15	23	23	14	26
Pump type, *n* (%)	2 Medtrum, 1 OmniPod 5, 2 MiniMed 780G, 1 Loop (Omnipod + Dexcom G6)	Medtrum	OmniPod 5	Medtrum	780G	780G	Open-Source AID
Previous Hajj experience	All first-time pilgrims	First time	First time	First time	First time	First time	First time
Self-reported glucose control during Hajj	Excellent (3), Very good (2), Not available (1)	Very good	Excellent	Excellent	Excellent	NA	Very good
Available pump/CGM data?	-	Yes	Yes	Yes	Yes	Yes	NA

Abbreviations: NA, not available.

**Table 2:** Changes in select CGM metrics, insulin doses, and rates of acute diabetes-related complications across pre-, during, and post-Hajj periods (*n* = 5).

	Pre-Hajj	During Hajj	Post-Hajj
Glucose CV, mean, %	35.6	32.66	33.1
GRI, mean	50.3	51.0	39.3 *
Insulin: Total daily dose (TDD), mean, units	47.57	44.93	42.74
Insulin: Basal (%)	48.52	49.08	49.42
Insulin: Bolus (%)	51.48	50.92	50.58
Severe hypoglycemia, *n* (%)	0 (0)	0 (0)	0 (0)
Diabetic ketoacidosis (DKA), *n* (%)	0 (0)	0 (0)	0 (0)
ER visit due to diabetes, *n* (%)	0 (0)	0 (0)	0 (0)
Hospitalization due to diabetes, *n* (%)	0 (0)	0 (0)	0 (0)

Abbreviations: CV, coefficient of variation; GRI, Glycemia Risk Index; DKA, diabetic ketoacidosis; ER, emergency room. * *p* < 0.05 comparing the post-Hajj to pre-Hajj.

**Table 3:** Summary of the experiences and feedback from AID users during Hajj.

Theme	Summary of Responses	Example Participant Quotes (Translated from Arabic)
Glucose Levels	Most participants reported stable glucose control throughout Hajj. Three participants described their control as excellent, and two as very good. No major complications occurred. One participant reported symptomatic hypoglycemia that required her to drink juice.	“My sugar remained stable throughout the pilgrimage.” “The pump made it easy to manage my glucose despite the long walking.”
Pump Use and Adjustments	Two participants adjusted their pump settings prior to Hajj. The rest did not need changes. All reported that using the pump during Hajj was easy.	“I didn’t need to change my settings.” “The use of pump during Hajj was very convenient with not challenges”.
Perceived Benefits	All participants emphasized peace of mind, flexibility, and confidence in performing rituals. They valued continuous insulin delivery and reduced fear of hypoglycemia.	“I felt safe and comfortable using the pump.” “It gave me peace of mind and helped me perform rituals confidently.” “It gave me peace of mind during the prolonged walking. I did not need to worry much about my sugar”. “I was worried about low sugar with walking but that did not happen”.
Challenges	Main difficulties included the heat, insulin storage, prolonged walking, and irregular meals. One participant experienced mild hypoglycemia, which was easily managed.	“It was hard to keep insulin cool in the heat. There were no enough coolers”. “The long walks are challenging”. “Irregular meals made glucose control harder.” “It was challenging to avoid low sugar. I had to carry snacks with me all the time”. “I had to eat frequent snacks throughout the day as opposed to fixed meals. I could not figure the proper doses for those snacks.”
Support and Preparation	All participants carried backup insulin and supplies. Preparation was key to success.	“The group I was with provided excellent medical support.” “I had all my supplies ready before travel.”
Current gaps and recommendations	Participants identified major gaps in awareness about insulin pumps and CGMs among the medical teams in Hajj.	“When I arrived to Arafat, my pump site failed, and I had no spare supplies. The GP physician said I didn’t need basal insulin since the CGM and pump were working”. “We need HCPs specialized in diabetes to be available and accessible during Hajj. It would be great to provide pilgrims with diabetes with digital wrist bands that contain the person’s medical history and is connected to the emergency medical teams in case of emergencies”. “Educate Hajj staff and teams about insulin pumps and CGMs.”
	All participants recommended that people with type 1 diabetes use an insulin pump during Hajj and beyond	
	One participant emphasized the importance of planning ahead and changing the insulin reservoir early, before insulin runs out.	

**Table 4:** Practical recommendations for clinicians and AID users performing Hajj.

	Recommendations
Pre-Hajj Preparation	AID users should undergo a comprehensive pre-Hajj evaluation by a specialized healthcare team 2–4 weeks before travel to Hajj. Review the pump/CGM report and optimize the AID settings. Create a backup plan for device failure (i.e., doses of multiple daily injection). Prepare extra supplies (insulin, pump infusion sets, reservoirs/cartridges/pods, CGM sensors, batteries, glucagon emergency kit, and blood glucose and ketone meters). Distribute supplies across multiple bags to reduce risk of loss or damage. Carry the insulin in a portable cooler at all times. Carry a medical report.
During Hajj	Activate the exercise/temporary target mode during prolonged walking or heat exposure. Monitor glucose levels frequently and act promptly. Keep fast-acting carbohydrates readily available to treat hypoglycemia. Avoid exposing insulin or device to direct sunlight or heat. Carry at least one full set of infusion sets and sensor supplies at all times. Monitor ketone levels whenever DKA is suspected or when glucose levels remain elevated despite corrective insulin doses.
Post-Hajj Follow-Up	Conduct post-Hajj reviews to assess glycemic outcomes and overall Hajj experience and adjust management plans. Use insights gained to refine future diabetes management strategies for Hajj.
Healthcare System Recommendations	Provide basic training for HCPs on insulin pumps and CGMs, including management of device failure. Establish specialized diabetes support units at Hajj healthcare facilities. Implement digital wristbands or medical ID systems linking pilgrims with diabetes to emergency services.

Abbreviations: CGM, continuous glucose monitoring; AID, automated insulin delivery; HCP, healthcare professional.

## Data Availability

The original contributions presented in this study are included in the article. Further inquiries can be directed to the corresponding author.
